# Low-coverage whole genome sequencing of diverse *Dioscorea bulbifera* accessions for plastome resource development, polymorphic nuclear SSR identification, and phylogenetic analyses

**DOI:** 10.3389/fpls.2024.1373297

**Published:** 2024-03-06

**Authors:** Ruisen Lu, Ke Hu, Xiaoqin Sun, Min Chen

**Affiliations:** ^1^Institute of Botany, Jiangsu Province and Chinese Academy of Sciences, Nanjing, China; ^2^Jiangsu Key Laboratory for the Research and Utilization of Plant Resources, Nanjing, China; ^3^Jiangsu Provincial Science and Technology Resources Coordination Platform (Agricultural Germplasm Resources) Germplasm Resources Nursery of Medicinal Plants, Nanjing, China

**Keywords:** *Dioscorea bulbifera*, plastome resources, polymorphic nSSRs, intraspecific phylogeny, molecular breeding

## Abstract

*Dioscorea bulbifera* (Dioscoreaceae), a versatile herbaceous climber native to Africa and Asia, holds significant nutritional and medicinal value. Despite extensive characterization and genetic variability analyses of African accessions, studies on the genetic variation of this species in China are limited. To address this gap, we conducted low-coverage whole genome sequencing on *D. bulbifera* accessions from diverse regions across mainland China and Taiwan island. Our initial investigation encompassed comprehensive comparative plastome analyses of these *D. bulbifera* accessions, and developing plastome resources (including plastome-derived repetitive sequences, SSRs, and divergent hotspots). We also explored polymorphic nuclear SSRs and elucidated the intraspecific phylogeny of these accessions. Comparative plastome analyses revealed that *D. bulbifera* plastomes exhibited a conserved quadripartite structure with minimal size variation mainly attributed to intergenic spacer regions, reinforcing prior observations of a high degree of conservation within a species. We identified 46 to 52 dispersed repeats and 151 to 163 plastome-derived SSRs, as well as highlighted eight key divergent hotspots in these *D. bulbifera* accessions. Furthermore, we developed 2731 high-quality candidate polymorphic nuclear SSRs for *D. bulbifera*. Intraspecific phylogenetic analysis revealed three distinct clades, where accessions from Southeast China formed a sister group to those from South China and Taiwan island, and collectively, these two clades formed a sister group to the remaining accessions, indicating potential regional genetic divergence. These findings not only contributed to the understanding of the genetic variation of *D. bulbifera*, but also offered valuable resources for future research, breeding efforts, and utilization of this economically important plant species.

## 1 Introduction

*Dioscorea bulbifera* L., commonly referred to as the air potato, air yam, bitter yam, cheeky yam, potato yam, is a dioecious herbaceous climber belonging to the yam family, Dioscoreaceae (Coursey, 1967; Kundu et al., 2021). This species is native to Africa and Asia, but has widely naturalized and is cultivated across various regions, including Central and South America, Nepal, China, the Americas, the West Indies, Pacific Islands, Southeast Asia, and even parts of Australia (Coursey, 1967; Guan et al., 2017; Kundu et al., 2021; Kuncari, 2022). This species is characterized by a twining stem with a sleek surface and alternately arranged vibrant green leaves in a broadly cordate shape (Burkill, 1960; Ding and Gilbert, 2000). The emergence of purplish-brown bulbils (aerial tubers) from leaf axils is particularly noteworthy, as these serve as the primary organ for asexual propagation of the species (Terauchi et al., 1991; Kundu et al., 2021). Additionally, the plant generates underground tubers that bear a resemblance to petite potatoes (Terauchi et al., 1991; Kundu et al., 2021).

*Dioscorea bulbifera* is a rich source of primary metabolites, encompassing carbohydrates, starch, sugars, proteins, lipids, vitamins, minerals, and fibers (Abara et al., 2011). Its tubers offer versatile culinary possibilities, being adaptable to roasting and cooking as a vegetable, providing sustenance for tribal communities during food crises (Dutta, 2015; Ojinnaka et al., 2017). Significantly, the presence of essential amino acids, such as threonine and phenylalanine, coupled with significant mineral content, notably iron, enhances its nutritional importance (Ezeocha et al., 2014; Otegbayo et al., 2018). Beyond its nutritional richness, *D. bulbifera* holds a profound place in traditional medicine serving as a purgative, anthelmintic, diuretic, rejuvenating tonic, and exhibiting aphrodisiac qualities (Kumar et al., 2017). In traditional Chinese medicine, *D. bulbifera* is employed to address conditions such as cough, pharyngitis, skin infections, piles, hemoptysis, and goiter (Guan et al., 2017). Recent studies have highlighted the potency of *D. bulbifera* against cancer, demonstrating its efficacy in inhibiting tumor growth in various cells, including colon and liver cancer (Guan et al., 2017).

The vast nutritional and medicinal benefits of *Dioscorea bulbifera* have triggered substantial characterization and genetic variability analyses in diverse regions like Brazil (Silva et al., 2016), Ethiopia (Beyene, 2013; Mulualem and Weldemichel, 2013), Nigeria (Jayeola and Oyebola, 2013), Uganda (Ikiriza et al., 2023), and West Africa (Osuagwu and Edem, 2020). These investigations have significantly propelled the advancement of breeding techniques aimed at enhancing its desirable traits for both food and medicinal purposes in these areas (Osuagwu and Edem, 2020). Nevertheless, despite the abundance of resources in China (Guan et al., 2017), the characterization and genetic variation analyses of this species lag far behind. It remains underutilized, marginalized, and less cultivated in China. Consequently, there exists an urgent necessity for comprehensive characterization, particularly at the molecular level, and genetic variation analysis of this plant in China.

Nowadays, the application of low-coverage whole genome sequencing has emerged as a cost-effective and efficient strategy for selectively capturing high-copy elements such as the plastome, ribosomal DNA, and SSRs across diverse plant species (Straub et al., 2012; Dodsworth, 2015; Lu et al., 2022). Among these molecular markers, whole plastome sequences, have demonstrated immense value in plant phylogenetic studies owing to their distinctive traits, including the absence of recombination, small effective population sizes, low nucleotide substitution rates, and typically uniparental inheritance (Birky et al, 1983). Additionally, conducting comparative plastomes analyses can facilitate the identification of regions with sequence variation, thereby aiding in accurate and rapid species discrimination—a critical element for the optimal utilization and conservation of plant species (Lu et al., 2021). More significantly, utilizing assembled nuclear sequences derived from low-coverage whole genome sequencing data has successfully enabled the extensive exploration of polymorphic nuclear SSRs (nSSRs) in non-model plant species (Liu et al., 2018, Liu et al., 2021), which play pivotal roles in population genetic analyses and marker-assisted selection (Kumar et al., 2015).

In this study, we conducted low-coverage whole genome sequencing of *Dioscoea bulbifera* accessions spanning diverse regions across mainland China and Taiwan island. Our objectives were to: (1) explore and compare *D. bulbifera* plastomes to unravel their evolutionary patterns; (2) pinpoint plastome-derived markers including repetitive sequences, plastomic SSRs, and divergent hotspots; (3) develop polymorphic nuclear SSRs using multiple assembled nuclear sequences; and (4) reconstruct the phylogenetic relationships among *D. bulbifera* accessions based on plastome data. These discoveries will not only broaden our understanding of the genetic variations within *D. bulbifera* accessions but also furnish essential genetic resources pivotal for advancing molecular characterization and commercial breeding schemes of this species.

## 2 Materials and methods

### 2.1 Plant materials, DNA extraction and genome sequencing

We gathered 10 *Dioscoea bulbifera* accessions from various regions across mainland China, spanning Anhui (AHLA), Fujian (FJZZ), Guangdong (GDSG), Guangxi (GXQZ), Henan (HeNXY), Hunan (HuNXX), Jiangsu (JSYX), Sichuan (SCEM), Zhejiang (ZJLS) provinces, along with Taiwan island (TWXB) (**Table 1**), for comprehensive analysis. For each accession, the pristine, fresh green leaves were harvested from a wild mature individual, and then preserved by desiccation with silica gel. Voucher specimens were deposited at the Herbarium of Institute of Botany, Jiangsu Province and the Chinese Academy of Sciences (NAS). Genomic DNA was then extracted from ~50 mg of silica-dried leaves using the DNAsecure Plant Kit (Tiangen Biotech, Beijing, China), with the purity and integrity of the isolated genomic DNA evaluated through agarose gel electrophoresis and spectrophotometric analysis.

**Table 1:** Summary of plastome characteristics of 10 *Dioscorea bulbifera* accessions.

Characteristics	AHLA	FJZZ	GDSG	GXQZ	HeNXY	HuNXX	JSYX	SCEM	TWXB	ZJLS
Locality	Lu’an, Anhui	Zhangzhou, Fujian	Shaoguan, Guangdong	Qinzhou, Guangxi	Xinyang, Henan	Xiangxi, Hunan	Yixing, Jiangsu	Emeishan, Sichuan	Xinbei, Taiwan	Lishui, Zhejiang
Clean Reads	45,979,402	51,380,484	37,387,226	40,006,520	43,568,546	39,480,040	49,054,050	45,186,582	53,141,458	41,480,186
Latitude (N)/ Longitude (E)	31°25′57″/ 116°8′31″	24°33′06″/ 117°20′07″	24°55′33″/ 113°01′22″	21°59′06″/ 108°42′16″	30°59′45″/ 116°04′49″	29°07′40″/ 110°27′48″	31°10′05″/ 119°40′55″	29°35′48″/ 103°22′13″	24°52′03″/ 121°24′51″	27°54′50″/ 118°55′23″
Total plastome length (bp)	153,074	153,002	153,099	153,002	152,970	153,093	153,074	153,093	153,002	153,074
LSC	83,225	83,152	83,249	83,152	83,120	83,240	83,225	83,240	83,152	83,225
SSC	18,851	18,852	18,852	18,852	18,852	18,855	18,851	18,855	18,852	18,851
IR	25,499	25,499	25,499	25,499	25,499	25,499	25,499	25,499	25,499	25,499
Total GC content (%)	37.0	37.0	37.0	37.0	37.0	37.0	37.0	37.0	37.0	37.0
LSC	34.8	34.8	34.8	34.8	34.8	34.8	34.8	34.8	34.8	34.8
SSC	30.8	30.8	30.8	30.8	30.8	30.8	30.8	30.8	30.8	30.8
IR	43.0	43.0	43.0	43.0	43.0	43.0	43.0	43.0	43.0	43.0
Total number of genes	113	113	113	113	113	113	113	113	113	113
PCGs	79	79	79	79	79	79	79	79	79	79
tRNA genes	30	30	30	30	30	30	30	30	30	30
rRNA genes	4	4	4	4	4	4	4	4	4	4
Duplicated genes	19	19	19	19	19	19	19	19	19	19

Approximately 1 µg genomic DNA was broken into small fragments using the Covaris E210 sonicator (Covaris Inc., MA, USA). Fragments were then size-selected selected by Agencourt AMPure XP-Medium kit (Thermo Fisher Scientific, USA) to attain sizes ranging from 200 to 400 bp. Following end repair, 3’adenylation, adaptor ligation, PCR amplification, and purification, the resulting double-stranded PCR products were transformed into single-stranded circular DNA (ssCir DNA), through heat denaturation and circularization using a splint oligo sequence. The ssCir DNA was formatted as the final library, and sequenced on DNBSEQ-T7 sequencing platform to generate 150-bp paired-end reads.

### 2.2 Plastome assembly and annotation

After preprocessing raw data with Trimmomatic v.0.36 (Bolger et al., 2014) to remove adaptor sequences, contamination, and low-quality reads, each accession yielded a total of 39,480,040–53,141,458 clean reads utilized for subsequent plastome assembly. *De novo* assembly of the plastome was executed using GetOrganelle v.1.7.6 (Jin et al., 2020), with recommended parameters: -R 15 -k 21,45,65,85,105 -F embplant_pt. Plastome sequences of all 10 accessions were assembled in complete circular sequences. Initial annotation was conducted with the MAFFT v.7 plugin (Katoh and Standley, 2013) integrated within Geneious Prime® 2022.0.1 (https://www.geneious.com), by aligning them with the previously published ones of *Dioscoea bulbifera* (MG805604) and *D. nipponica* (OQ525997) as references. Reference annotations were then transferred to these newly assembled plastomes, followed by meticulous manual verification to ensure precision of exon/intron boundaries and accurate start/stop codon placement. To present comprehensive insights of *D. bulbifera* plastomes, high-resolution circular plastome maps were generated using the web-based tool OrganellarGenomeDRAW (OGDRAW) v.1.3.1 (Greiner et al., 2019).

### 2.3 Whole plastome sequence comparison

The mVISTA program (http://genome.lbl.gov/vista/mvista/submit.shtml) was employed to assess the structural resemblance of complete plastome sequences among *Dioscoea bulbifera* accessions, with the annotation from the AHLA plastome sequence serving as the reference. Plastome sequences were aligned using the Shuffle-LAGAN mode with default parameters (Brudno et al., 2003), and the resulting alignments were displayed using the VISTA program (Frazer et al., 2004). Additionally, to detect potential expansions or contractions in the inverted repeat (IR) regions within *D. bulbifera* plastomes, a comparison and visualization of the four junctions between the inverted repeat (IR) and the large single copy (LSC)/small single copy (SSC) regions were conducted using IRscope (https://irscope.shinyapps.io/irapp/, Amiryousefi et al, 2008).

### 2.4 Plastome-derived markers development

Repetitive sequences, comprising forward (direct), reverse, complement, and palindromic repeats within *Dioscoea bulbifera* plastomes, were identified through the online tool REPuter (Kurtz et al., 2001). The parameters for repetitive sequences identification involved a minimum repeat size of 30 bp, a sequence identity of at least 90%, and a hamming distance of 3. Simple sequence repeats (SSRs) within the 10 *D. bulbifera* plastome sequences were identified using the MISA-web application (Beier et al., 2017). The SSR search criteria specified a minimum of 10 repeat units for mononucleotide SSRs, 5 for dinucleotide SSRs, 4 for trinucleotide SSRs, and 3 for tetra-, penta-, and hexanucleotide SSRs, respectively.

For an in-depth exploration of divergent hotspots within *Dioscoea bulbifera* plastomes, the 10 newly assembled plastome sequences were aligned using MAFFT v.7 (Katoh and Standley, 2013) in Geneious Prime® 2022.0.1. Regions within this alignment, including protein coding areas, intergenic spacers, and introns, displaying a total mutation count above 0 and an aligned length exceeding 200 bp, were systematically extracted from the alignment matrix. Subsequently, the nucleotide diversity (π) of these extracted regions was computed using DnaSP v.6.12.03 (Rozas et al., 2017).

### 2.5 Polymorphic nuclear SSRs identification

Low-coverage whole genome sequencing data from three geographically distinct *Dioscoea bulbifera* accessions (AHLA, SCEM, and TWXB) were employed to develop polymorphic nuclear SSR markers. Clean reads of these accessions were aligned to the genome sequences of *D. alata* (Bredeson et al., 2022) and *D. zingiberensis* (Li et al., 2022) to exclude mitochondria and chloroplast reads using BWA-MEM v.0.7.17 (Li, 2013). The resulting alignment files were sorted and converted into Binary Alignment/Map (BAM) format using SAMtools v.1.9 (Li et al., 2009). Subsequently, BAM files were transformed into FastQ files with SAMtools bam2fq. The obtained reads were then *de novo* assembled into contigs using CLC Genomics Workbench v.23.0.4 (CLC bio, Aarhus, Denmark) with default settings. Utilizing these assembled nuclear sequences, CandiSSR (Xia et al., 2016) was employed to identify polymorphic nuclear SSRs (nSSRs) within *D. bulbifera*, using the default parameters except for specifying a flanking sequence length of 200 bp.

### 2.6 Phylogenetic analyses within *Dioscoea bulbifera*

Phylogenetic analyses were performed using two datasets: complete plastome sequences and 79 protein coding genes shared across all 10 *Dioscoea bulbifera* accessions (**Table 1**), employing *D. nipponica* (OQ525997), *D. elephantipes* (EF380353) and *D. alata* (OP787123) as outgroups, based on Noda et al. (2020). For the complete plastome dataset, both maximum likelihood (ML) and Bayesian inference (BI) analyses were conducted employing two partitioning scenarios: (1) unpartitioned, and (2) partitioned by each gene and intergenic region (265 partitions). In contrast, the protein coding gene dataset underwent four partitioning scenarios: (1) unpartitioned, (2) partitioned by codon position (three partitions), (3) partitioned by each gene (79 partitions), and (4) partitioned by PartitionFinder v.2.1.1 (Lanfear et al., 2017) (19 partitions). Alignments of both complete plastome sequences and protein coding sequences were executed using the MAFFT v.7 plugin (Katoh and Standley, 2013) within Geneious Prime® 2022.0.1. Except for the partition scenario determined through PartitionFinder, the optimal substitution model was obtained using PartitionFinder, while the remaining partition schemes selected GTR + I + G as the optimal substitution based on the Akaike Information Criterion (AIC) computed by jModelTest v.2.1.4 (Darriba et al., 2012). Maximum Likelihood (ML) analyses were carried out using RAxML v.8.2.12 (Stamatakis, 2014) via the CIPRES Science Gateway v.3.3 (http://www.phylo.org/portal2/), utilizing 1000 bootstrap replications. Bayesian Inference (BI) analyses were conducted using MrBayes v.3.2.7 (Ronquist et al., 2012), utilizing Markov Chain Monte Carlo (MCMC) runs for 1 × 10^6^ generations with a sampling frequency of 1000 trees. The initial 1000 trees were discarded as ‘burn-in’, and the remaining trees were employed to generate a majority-rule consensus tree and estimate posterior probabilities (PPs).

## 3 Results

### 3.1 Plastome characteristics of *Dioscoea bulbifera*

**Figure 1:**
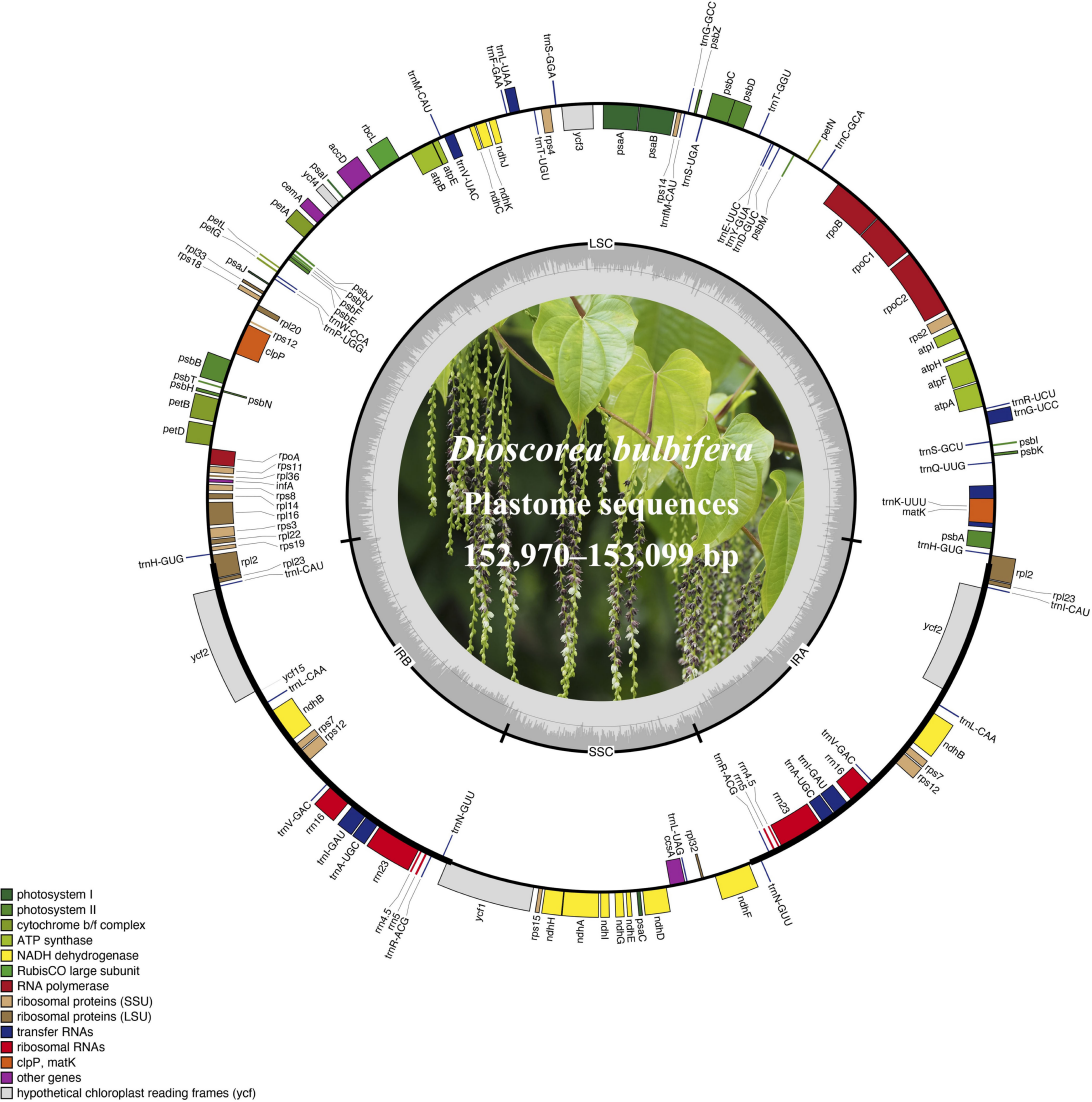
The plastome maps of *Dioscorea bulbifera* accessions. Genes located on the outer circle are transcribed clockwise, while those on the inner circle are transcribed counter-clockwise. Functional categories are distinguished by color-coded genes. The inner ring showcases darker grey representing GC content and lighter grey representing AT content. Additionally, a plant photograph of *D. bulbifera* is featured within the inner circle.

The plastome sizes exhibited narrow variation among these 10 *Dioscoea bulbifera* accessions: GDSG displayed a length of 153,099 bp, HuNXX and SCEM had a size of 153,093 bp, AHLA, JSYX, and ZJLS shared a size of 153,074 bp, FJZZ, GXQZ, and TWXB showcased a size of 153,002 bp, while HeNXY exhibited a size of 152,970 bp (**Figure 1**; **Table 1**). Each of these *D. bulbifera* plastomes maintained the typical circular quadripartite structure, comprising a pair of inverted repeat (IR) regions (25,499 bp) separated by a large single copy (LSC) region ranging between 83,120–83,249 bp, and a small single copy (SSC) region varying from 18,851–18,855 bp. The GC content across the entire plastome sequence (37.0%), as well as in the LSC (34.8%), SSC (30.8%), and IR (43.0%) regions, remained consistent among all 10 *D. bulbifera* accessions.

All 10 *Dioscoea bulbifera* plastomes shared an exact set of 113 unique genes, encompassing 79 protein-coding genes (PCGs), 30 transfer RNA (tRNA) genes, and four ribosomal RNA (rRNA) genes (**Figure 1**; **Table 1**). Out of these, 19 genes (seven PCGs, eight tRNA genes, and all four rRNA) were duplicated within the inverted repeats (IRs), resulting in a cumulative count of 132 genes (**Table 1**; **Supplementary Table S1**). Among these unique genes, eight PCGs and six tRNAs contained a single intron, while three PCGs harbored two introns each (see details in **Supplementary Table S1**). An intact gene encoding initiation factor IF1 (*infA*) was identified, while the *rps16* gene was independently lost in *D. bulbifera* plastomes (**Figure 1**).

### 3.2 Plastome comparison within *Dioscoea bulbifera*

**Figure 2:**
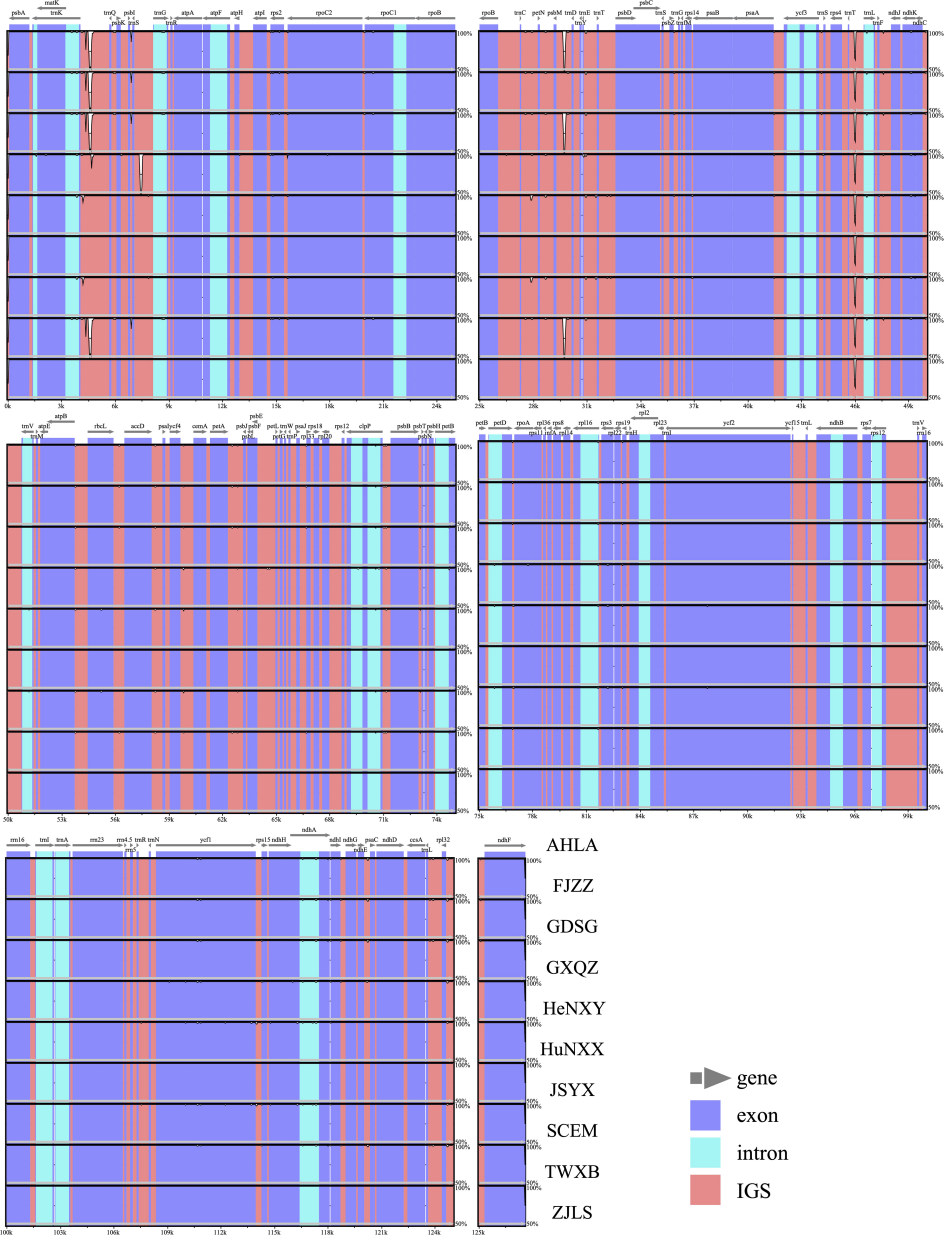
Sequence similarity plots among *Dioscorea bulbifera* plastomes, using the accession AHLA as a reference. Annotated genes are shown along the top. The vertical scale indicates percent identity, ranging from 50% to 100%. Genome regions are color-coded, distinguishing between exons, introns, and intergenic spacers (IGS).

Using the accession AHLA as the reference, mVISTA results indicated high sequence similarity among these *Dioscoea bulbifera* plastomes, especially in the IR regions (**Figure 2**). Moreover, the coding regions demonstrated notably higher similarity levels compared to non-coding regions, including introns and intergenic spacers. Notably, the intergenic spacers, specifically *trnK*–*trnQ* and *psbM*–*trnD*, displayed the lowest sequence similarity (**Figure 2**). Within the *trnK*–*trnQ* region, AHLA, JSYX, HeNXY, HuNXX, SCEM, and ZJLS showed a 24 bp gap compared to FJZZ, GDSC, GXQZ, and TWXB. Similarly, in the *psbM*–*trnD* region, FJZZ, GXQZ, and TWXB presented a more substantial 96 bp gap compared to the other accessions.

**Figure 3:**
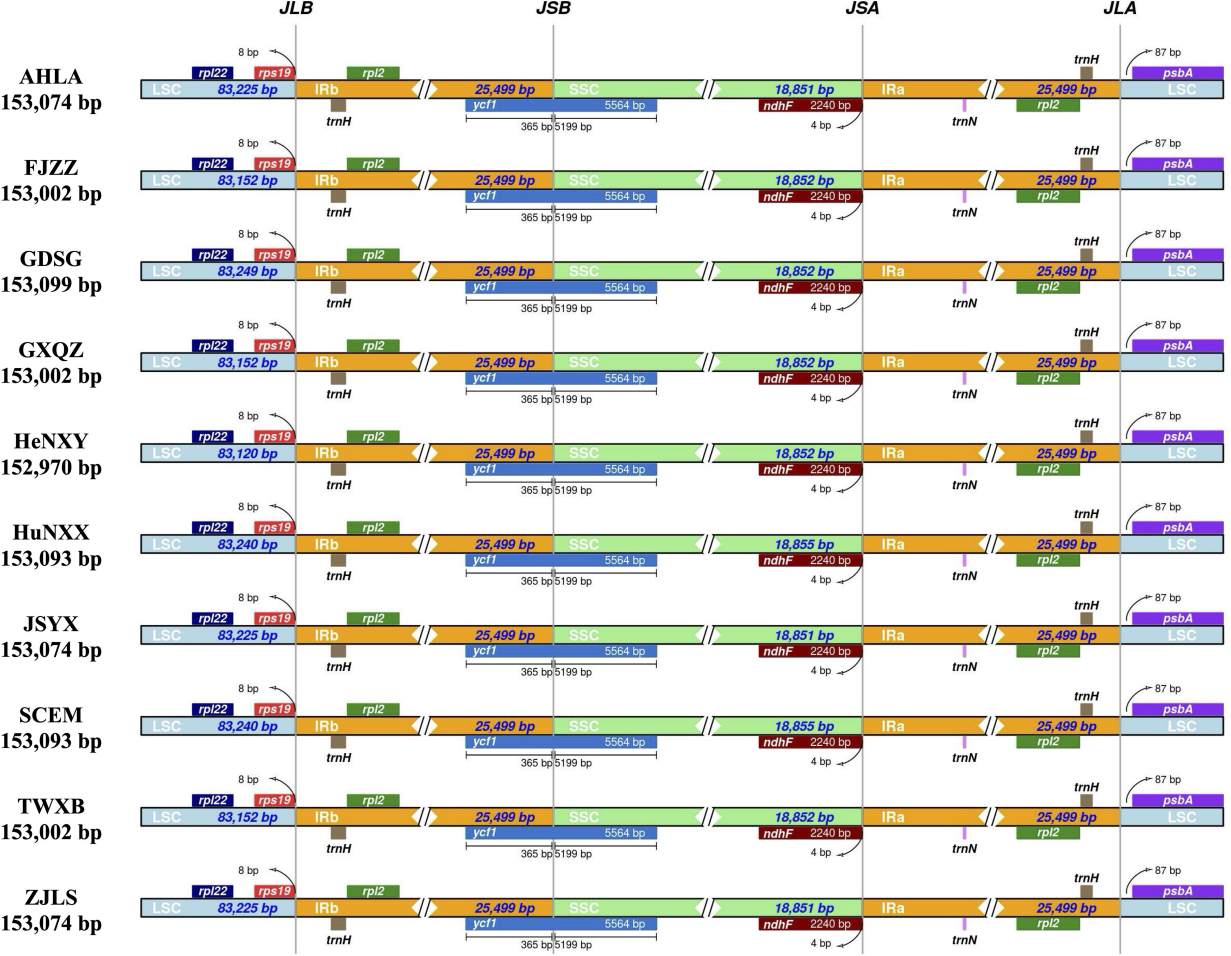
Comparison of inverted repeat/single copy (IR/SC) junction regions in *Dioscorea bulbifera* plastomes.

Comparative analysis of IR/SC junctions underscored the stability of *Dioscoea bulbifera* plastomes, revealing no expansion or contraction in the IR regions among these accessions (**Figure 3**). Across these 10 plastomes, the LSC/IRa junction (JLA) was situated in the *psbA*–*trnH* intergenic spacer region, 87 bp away from the adjacent gene *psbA*. Concurrently, the LSC/IRb junction (JLB) was positioned within the intergenic spacer of *rps19*–*trnH*, maintaining an 8 bp distance from the *rps19* gene (**Figure 3**). Notably, the *ycf1* gene traversed the LSC/IRb junction (JLB), maintaining a consistent length of 365 bp within the IRb region and extending to 5199 bp in the SSC region. Meanwhile, the *ndhF* gene was completely located in the *SSC* region, merely 4 bp away from the SSC/IRa junction (JSA) (**Figure 3**).

### 3.3 Plastome-derived markers of *Dioscoea bulbifera*

**Figure 4:**
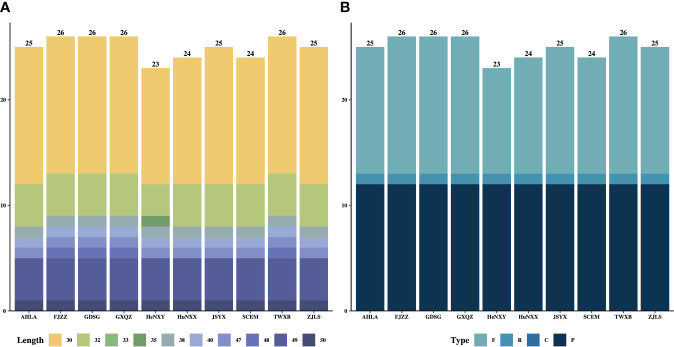
The **(A)** lengths and **(B)** types of dispersed repeats in the 10 *Dioscorea bulbifera* plastomes.

The types and lengths of dispersed repeats, including forward (direct), reverse, complement, and palindromic repeats, as well as simple sequence repeats (SSRs) were detected and analyzed within these 10 *Dioscoea bulbifera* plastomes. A total of 250 dispersed repeats were detected across all 10 *D. bulbifera* plastomes, comprising three repeat types: forward (120), reverse (10) and palindromic (120) repeats (**Figure 4A**). Among all 10 plastomes, FJZZ, GDSG, GXQZ and TWXB displayed the highest count of repeats (total: 26, forward: 13, reverse: 1, and palindromic: 12), followed by AHLA, JSYX and ZJLS (total: 25, forward: 12, reverse: 1, and palindromic: 12), and HuNXX and SCEM (total: 24, forward: 11, reverse: 1, and palindromic: 12), while HeNXY exhibited the fewest (total: 23, forward: 10, reverse: 1, and palindromic: 12) (**Figure 4A**). Across each *D. bulbifera* plastome, a substantial majority of repeats (61.5% in HeNXY to 76.0% in AHLA, JSYX, and ZJLS) ranged in size between 30 and 40 bp (**Figure 4B**).

**Figure 5:**
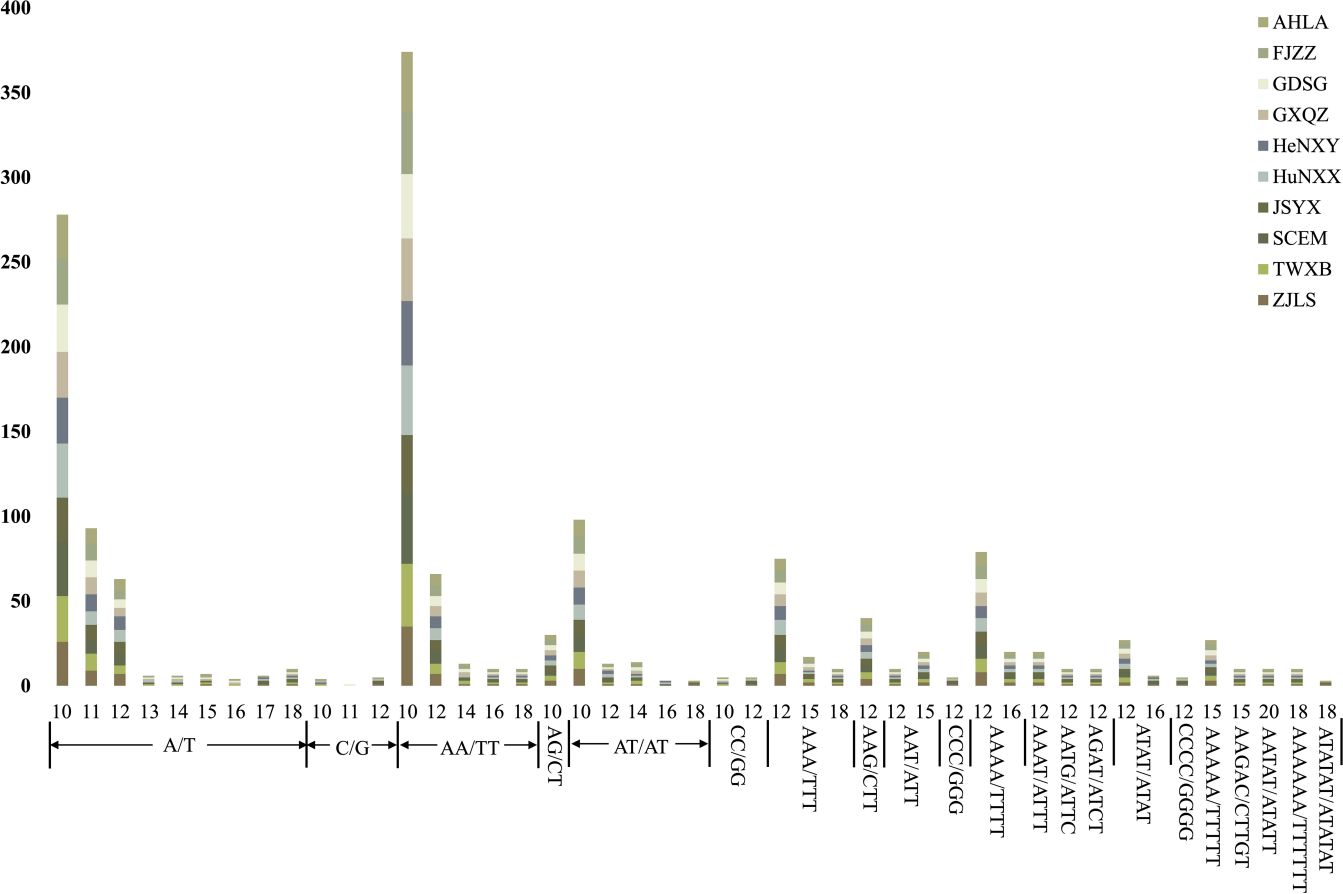
The lengths and motif types of plastome-derived SSRs in the 10 *Dioscorea bulbifera* accessions.

The count of plastome-derived SSRs within the *Dioscoea bulbifera* plastomes varied from 151 (AHLA, JSYX, and ZJLS) to 163 (HuNXX and SCEM) (**Figure 5**; **Supplementary Table S2**). Among these, dinucleotides emerged as the most prevalent SSR type, ranging from 62 in AHLA, JSYX, and ZJLS to 68 in HuNXX and SCEM. Following dinucleotides were mononucleotides, which varied from 46 in AHLA, JSYX, and ZJLS to 52 in HuNXX and SCEM. Trinucleotides and tetranucleotides exhibited a same pattern and were next in frequency, ranging from 17 in FJZZ, GDSG, GXQZ, HeNXY, and TWXB to 19 in HuNXX and SCEM. Conversely, pentanucleotides were observed in only 4 instances (HeNXY, HuNXX, SCEM) or 5 in the remaining accessions, while hexanucleotides were least common, occurring once in FJZZ, GDSG, GXQZ, HeNXY, HuNXX, SCEM, and TWXB, or twice in the other accessions, (**Figure 5**; **Supplementary Table S2**). Among the motifs in the SSRs, A/T and AA/TT were the most frequently occurring motifs, followed by AT/AT, AAA/TTT and AAAA/TTTT, while the remaining types appeared relatively infrequently (**Figure 5**; **Supplementary Table S2**). Moreover, a specific set of at least eight plastome-derived SSRs—(A/T)_16,17_, (C/G)_10,11,12_, (AT/AT)_6,7_, (AT/AT)_8,9_, (CC/GG)_5,6_, (CCC/GGG)_4_, (ATAT/ATAT)_3_, (CGCG/CGCG)_3_, (ATATAT/ATATAT)_3_ could effectively distinguish these *D. bulbifera* accessions into two to three distinct groups (**Supplementary Table S2**).

**Figure 6:**
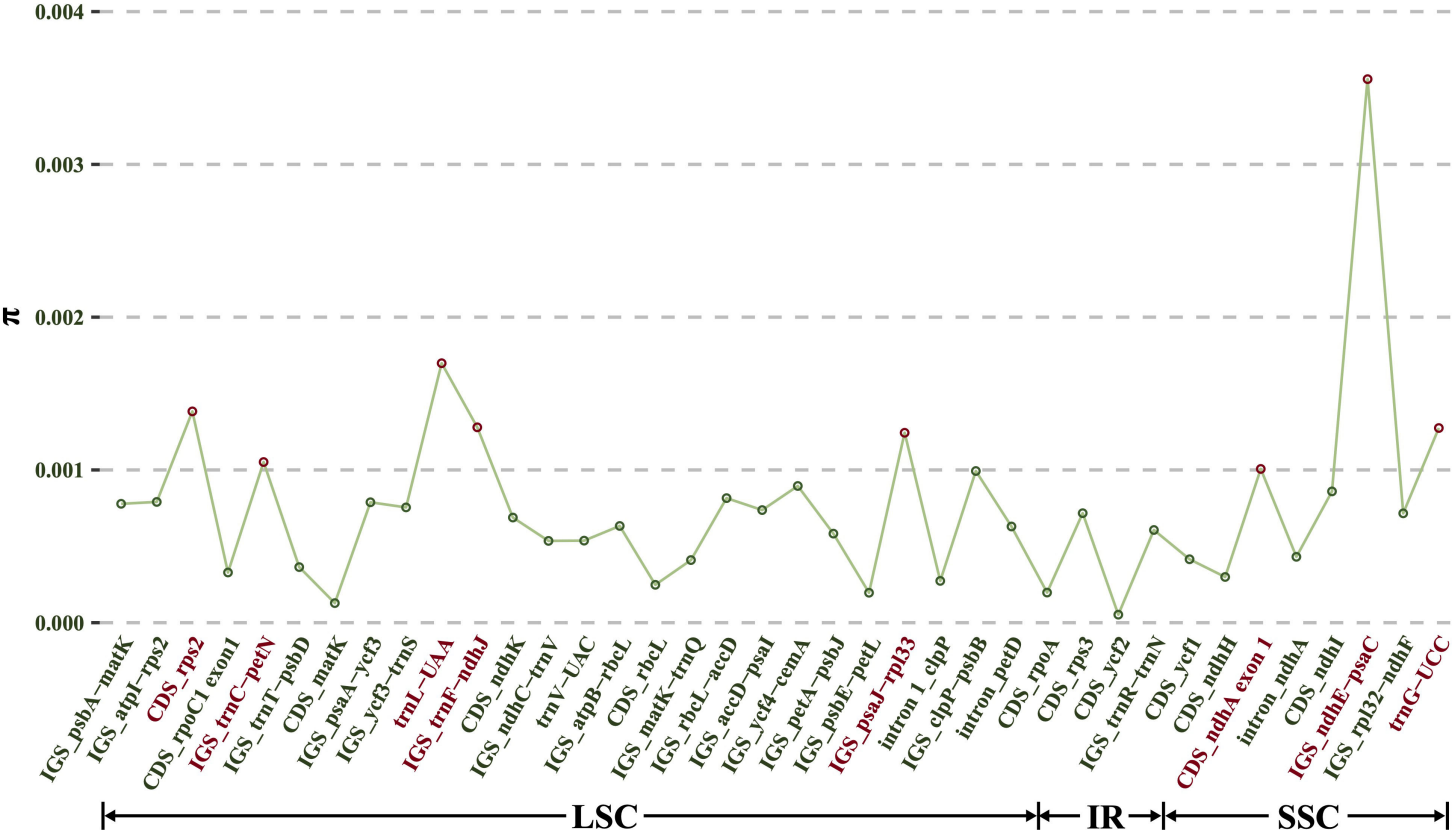
Nucleotide variability (π) values of 38 regions (12 CDS, 20 IGS, three introns and three tRNAs) extracted from the alignment matrix of 10 *Dioscorea bulbifera* plastomes.

Although a total of 133 regions (62 CDS, 52 IGS, 13 introns and 6 tRNAs) showed an aligned length exceeding 200 bp, only 38 regions (12 CDS, 20 IGS, 3 introns and 3 tRNAs) had a mutation count greater than zero. Consequently, these 38 regions were selected from the alignment of all 10 *Dioscoea bulbifera* plastomes to identify divergent hotspots (**Figure 6**). These 38 regions displayed π values ranging from 0.000053 (CDS *rpoC2*) to 0.0036 (IGS *ndhE*–*psaC*) (**Figure 6**). Among protein-coding regions (CDS), π values for each region ranged from 0.000053 (*rpoC2*) to 0.0014 (*rps2*), with only two CDS regions (*rps2* and exon 1 of *ndhA*) exhibiting notably high values (π > 0.001). For the non-coding regions (IGS, introns and tRNAs), *π* values ranged from 0.00020 (IGS *psbE*–*petL*) to 0.0036 (IGS *ndhE*–*psaC*). The top six most variable non-coding regions (π > 0.001) were IGS *ndhE*–*psaC*, *trnL*-*UAA*, IGS *trnF*–*ndhJ*, *trnG*-*UCC*, IGS *psaJ*–*rpl33*, and IGS *trnC*–*petN* (**Figure 6**). These six non-protein-coding regions in conjunction with the two protein-coding regions showed significant potential as highly informative molecular markers for *D. bulbifera*.

### 3.4 Polymorphic nuclear SSRs for *Dioscoea bulbifera*

Utilizing low-coverage whole-genome sequencing data, a total of 159,233, 164,875, and 192,193 nuclear contigs were generated for AHLA, SCEM, and TWXB, respectively, each having N50 lengths of 1,629 bp, 1,595 bp, and 1,650 bp. Based on these nuclear contigs, a pool of 2731 candidate polymorphic nSSRs (PolynSSRs) was identified for *Dioscoea bulbifera* (**Supplementary Table S3**). The average similarity of the flanking sequences of these polymorphic nSSRs was 0.96, with 71.95% (1965/2731) exhibiting a similarity above 0.98, indicating their high transferability across *D. bulbifera* accessions. Subsequent filtration, eliminating low-quality PolynSSRs with transferability (similarity) < 95% and a missing rate (MR) ≥ 0.5, resulted in a collection of 2433 high-quality candidate PolynSSRs (**Supplementary Table S3**). Out of these, 2331 high-quality PolynSSRs could be designed for primers, encompassing 95.81% of the total (**Supplementary Table S3**). Within this set of high-quality candidate PolynSSRs, tetranucleotide repeats comprised the majority at 1041 (42.79%), followed by tri-, penta-, and hexanucleotide repeats, accounting for 32.71%, 12.33%, and 12.17% of the total, respectively (**Supplementary Table S3**).

### 3.5 Intraspecific phylogeny of *Dioscoea bulbifera*

**Figure 7:**
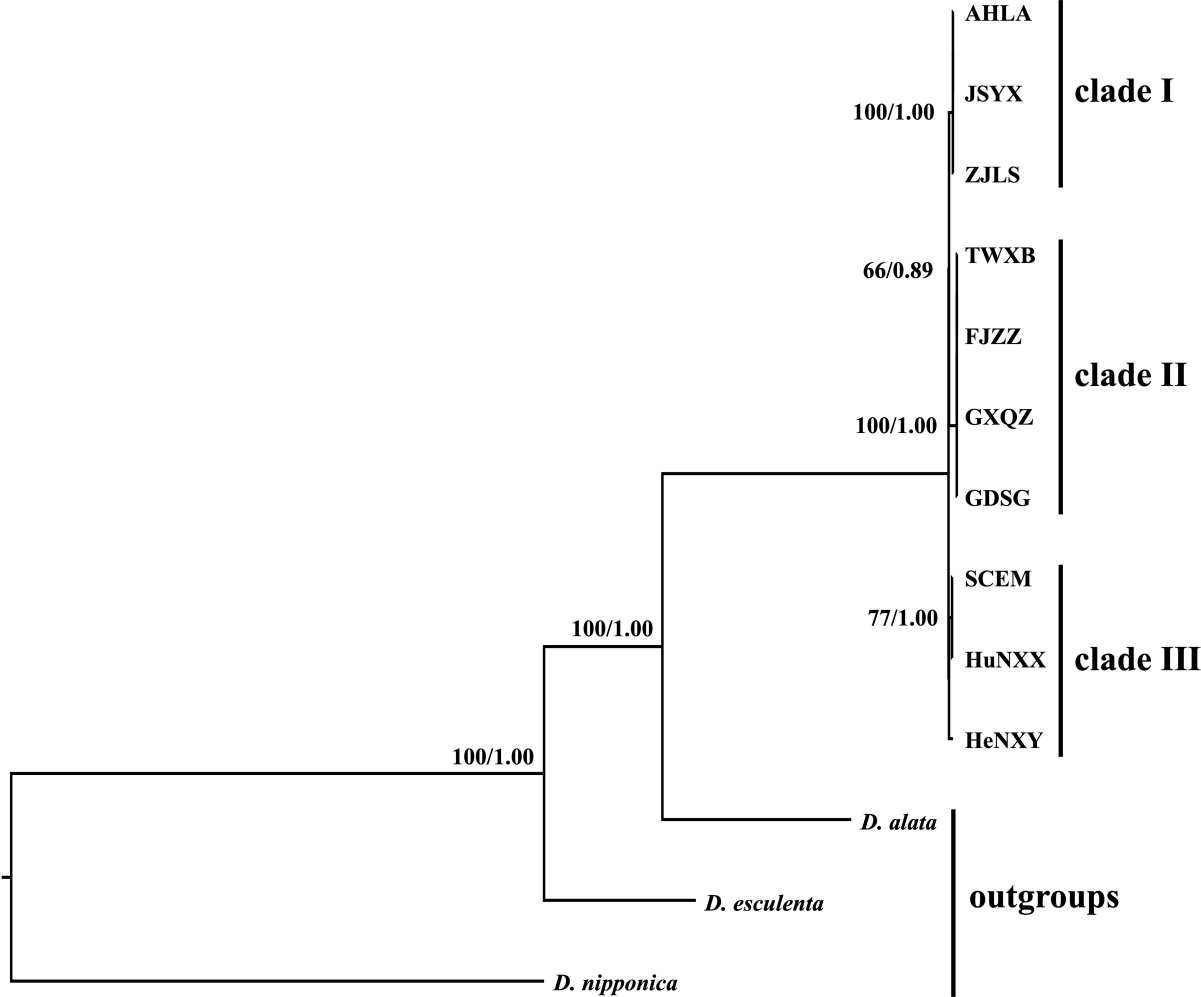
Intraspecific phylogenetic relationships among the 10 *Dioscorea bulbifera* accessions originating from different regions across mainland China and Taiwan island, inferred from the methods of maximum likelihood (ML) and Bayesian inference (BI). The ML bootstrap values/BI posterior probabilities are displayed above the lines.

Both the ML and BI analyses, employing complete plastome sequences and 79 shared protein coding genes under different partitioning strategies, yielded identical tree topologies. Consequently, only the phylogenetic trees based on complete plastome sequences are presented here (**Figure 7**). Phylogenetic analyses revealed three main distinct clades within these *Dioscoea bulbifera* accessions. Specifically, AHLA, ZJLS, and JSYX from Southeast China formed a distinctive monophyletic clade (Clade I), that is sister to the Clade II encompassing accessions from South China (FJZZ, GDSG, and GXQZ) and TWXB from Taiwan island. These two clades collectively form a sister group to the remaining accessions (HeNXY, HuNXX, and SCEM) (Clade III) (**Figure 7**).

## 4 Discussion

### 4.1 Plastome characteristics and evolution of *Dioscoea bulbifera*

The comprehensive exploration of 10 *Dioscoea bulbifera* plastomes from diverse geographic regions across mainland China and Taiwan island unveiled intriguing insights into the plastome structure, genetic composition, and variation of this species. Across all 10 accessions, the plastomes maintained a conserved quadripartite structure, with minimal size variation predominantly residing within the single copy regions (**Figure 2**; **Table 1**). The consistency in gene content (including unique and duplicated genes), gene order, and GC content of these *D. bulbifera* plastomes reinforced earlier findings that highlighted a high degree of conservation among plastomes within a species in terms of structure, gene composition, and gene order synteny (Muraguri et al., 2020; Lu et al., 2022). The absence of the *rps16* gene in *D. bulbifera* plastomes corroborated our prior observation that this gene may be absent across *Dioscorea* clades, excluding the *Stenophora* clade (= *D*. sect. *Stenophora*) (Jansen et al., 2007; Hu et al., 2023a). Conversely, the intact presence of the initiation factor IF1 gene (*infA*) in *D. bulbifera* plastomes aligned with its occurrence in other *Dioscorea* species (Lu et al., 2023; Hu et al., 2023a). Notably, among monocots, the depletion of *infA* genes is particularly concentrated (>70%) in Alismatales, Commelinales, Liliales, Pandanales, with minimal loss occurrences of 7.69% observed in Dioscoreales, including 12.50% within Dioscoreaceae (Lu et al., 2021).

Plastome size variations typically arise from two primary factors: i) the dynamic changes in the junctions between the inverted repeat (IR) and single copy (SC) regions (Kim and Lee, 2004; Lu et al., 2016), and ii) the variability of gene spacer regions, and the presence or absence of genes and introns (Jansen et al., 2007; Li et al., 2021). Our investigation of IR/SC junctions across *Dioscoea bulbifera* accessions unveiled a consistent structural configuration without observable expansions or contractions (**Figure 3**). Considering the conserved nature of gene content and structure, it appeared that differences in plastome sizes among these accessions mainly result from alterations in gene spacer regions. Significantly, the mVISTA analysis underscored that specific intergenic spacers, particularly *trnK*–*trnQ* and *psbM*–*trnD*, exhibited low sequence similarity, displaying gaps among the identified clades (**Figure 2**). This observation, coupled with our previous studies on plastomes of *D. alata* (Lu et al., 2023) and *D. nipponica* (Hu et al., 2023b), suggested that variations in intergenic spacers, particularly *trnK*–*trnQ*, could be contributing to the diversity in plastome sizes within individual *Dioscorea* species. However, further investigation is warranted to comprehensively understand these variations.

### 4.2 Molecular markers for *Dioscoea bulbifera*

The recognition of the importance of conserving medicinal plants, enhancing cultivars with desirable traits, and comprehending germplasm diversity has witnessed significant growth in recent years (Baruah et al., 2017; Marakli, 2018). This growing emphasis has led to the utilization of various molecular markers that offer elaborate genomic insights surpassing the capabilities of phenotypic methods (Marakli, 2018). Plastome-derived markers have emerged as valuable assets, enabling the identification of germplasm resources and contributing to their conservation and breeding efforts (Daniell et al., 2016). Despite the high conservation of plastome sequences within *Dioscoea bulbifera*, nucleotide substitutions, SSRs, and indels could serve as valuable markers to elucidate the genetic diversity and guide molecular breeding of this medically important plant (Li et al., 2020). In this study, we successfully identified a remarkable array of plastome-derived SSRs, ranging from 151 (AHLA, JSYX, and ZJLS) to 163 (HuNXX and SCEM) (**Figure 5**; **Supplementary Table S2**), and revealed at least eight potentially polymorphic SSRs, highlighting their utility in marker-assisted studies and population genetics.

Previous phylogenetic studies in *Disocorea* primarily relied on *matK*, *rbcL*, and *trnL*–*F* genes, which often lacked sufficient phylogenetic resolution within closely related species and within a single species (Hu et al., 2023a). Recent comparative plastome studies have emphasized the concentration of divergent hotspot regions in non-protein-coding areas across *Dioscorea* species. Notable examples included six IGS regions (i.e., *ndhD*–*ccsA*, *petA*–*psbJ*, *psbZ*–*trnG*, *rpl32*–*ndhF*, *trnD*–*trnY*, and *trnL*–*rpl32*) and the *rps16* intron sequence emerged as potential molecular markers for species within *D*. sect. *Stenophora* (Hu et al., 2023a). Similarly, regions such as *ndhD*–*ccsA*, *trnC*–*petN*, and *trnL*–*rpl32* were identified as potential DNA barcodes for species within *D*. sect. *Enantiophyllum* (Lu et al., 2023). Notably, regions like three intergenic spacers (*rps16*–*trnQ*, *trnE*–*trnT*, and *trnL*–*rpl32*) and two intron regions (intron 1 of *clpP* and intron *trnG*) promised substantial insights into assessing intraspecific genetic variability of *D. nipponica* (Hu et al., 2023b). Given these findings, further exploration of molecular markers, particularly in non-coding regions, becomes imperative in *Dioscorea* species. The comparative analysis across 10 *D. bulbifera* plastomes unveiled four IGS regions (*ndhE*–*psaC*, *trnF*–*ndhJ*, *psaJ*–*rpl33*, and *trnC*–*petN*), two tRNA regions (*trnL*-*UAA*, *trnG*-UCC*)*, and two CDS regions (*rps2* and exon 1 of *ndhA*) with notably high values of nucleotide diversity (**Figure 6**), holding substantial promise for population genetic and intraspecific phylogenetic studies of *D. bulbifera*.

Nuclear SSR markers (nSSRs) have demonstrated their value in diverse applications, including population genetic analyses, cultivar and germplasm identification, and marker-assisted selection, due to their high polymorphism and co-dominant inheritance in a Mendelian fashion (Kaldate et al., 2017). Recent advancements in sequencing technologies and bioinformatic analyses have opened an unprecedented window for identifying high-quality, polymorphic nuclear SSR markers in non-model organisms, offering effective results within optimized cost and time frames (Xia et al., 2016). In this study, a significant discovery of 2433 high-quality candidate PolynSSRs was made (**Supplementary Table S3**), providing potent tools for conducting population genetic studies of *Dioscoea bulbifera*. In summary, the identified intraspecific plastome-derived and nuclear markers could offer complementary insights into the genetic structure, differentiation, and gene flow among *D. bulbifera* populations, being crucial for their conservation and efficient management. Furthermore, these markers can be used to develop genetic maps and conduct marker assisted breeding.

### 4.3 Phylogenetic relationships of *Dioscoea bulbifera*

Nowadays, the utilization of whole plastome sequences has become widespread in elucidating the phylogenetic relationships among plant species (Lu et al., 2016; Hu et al., 2023b). Within the *Dioscorea* genus, the application of whole plastome sequences has significantly clarified previously ambiguous phylogenetic aspects in certain taxa. For instance, Magwé-Tindo et al. (2018) utilized whole plastomes to construct a robust and well-supported phylogenetic tree of West African *Dioscorea* species, revealing six monophyletic groups within them. Additionally, Hu et al. (2023a) conducted phylogenetic analyses for *D*. sect. *Stenophora* using plastome sequences, suggesting that *D. biformifolia* and *D. banzhuana* represent successive sister species to the remaining *Stenophora* species. Despite these advancements, limited research has explored intraspecific variation and phylogeny of *Dioscorea* species, using whole plastome data. In this study, phylogenetic analyses based on plastome sequences delineated three distinct clades among *D. bulbifera* accessions originating from diverse regions across mainland China and Taiwan island (**Figure 7**). The identification of three distinct clades implied potential genetic divergence among populations from different geographic regions. It is noteworthy that accession TWXB unexpectedly clustered with accessions from southern mainland China (FJZZ, GDSG, and GXQZ), displaying no mutations in their plastomes. This finding is surprising given that Taiwan Island became isolated around 10,000 years ago due to rising sea levels (Voris, 2000), leading to disrupted gene flow in many species through the formation of the Taiwan Strait (Lin et al., 2014). One plausible explanation for these results is that glaciations may have caused lowered sea levels, facilitating dispersal between Taiwan and mainland China and thereby obscuring the genetic endemism of Taiwanese accessions (Qu et al., 2015).

Overall, these findings underscored the significance of plastome phylogenomics in resolving intraspecific variation and phylogenetic relationships within *Dioscoea bulbifera*. Moving forward, it is imperative to acquire additional plastomes from *D. bulbifera* accessions in tropical Asia, Northern Australia, America, and sub-Saharan Africa (Kundu et al., 2021). This expansive dataset will provide a comprehensive perspective on the evolutionary relationships and processes of *D. bulbifera*, laying a robust foundation for further exploration of this economically significant species.

## 5 Conclusions

In conclusion, this study presented a comprehensive analysis of *Dioscorea bulbifera*, a versatile herbaceous climber with substantial nutritional and medicinal importance, through low-coverage whole genome sequencing. The investigation covered diverse accessions from mainland China and Taiwan, shedding light on the genetic variation within this species. Comparative plastome analysis revealed conserved structural features across accessions, with variations mainly attributed to intergenic spacer regions. The identification of plastome-derived markers, including dispersed repeats, SSRs, and divergent hotspots, along with high-quality polymorphic nuclear SSRs, provided valuable tools for population genetic studies and molecular breeding of *D. bulbifera*. The phylogenetic analysis revealed three distinct clades in these *D. bulbifera* accessions, indicating potential genetic divergence among populations from different geographic regions. Overall, this study not only addressed the existing gap in genetic variation studies of *D. bulbifera* in China but also laid the groundwork for further exploration and utilization of this valuable plant species.

## Data availability statement

The datasets presented in this study can be found in online repositories. The names of the repository/repositories and accession number(s) can be found below: All newly generated plastome sequences were deposited in GenBank (accession numbers: PP130724-PP130733). The low-coverage whole genome sequencing data of 10 Dioscorea bulbifera accessions generated in this study have been submitted to the NCBI SRA database (https://www.ncbi.nlm.nih.gov/sra/), under accession numbers: SRR27556260–SRR27556269.

## Author contributions

RL: Software, Writing – original draft. KH: Software, Writing – original draft. XS: Resources, Writing – review & editing. MC: Conceptualization, Funding acquisition, Resources, Writing – review & editing.
